# Influence of SARS-CoV-2 Variant B.1.1.7, Vaccination, and Public Health Measures on the Spread of SARS-CoV-2

**DOI:** 10.3390/v13050898

**Published:** 2021-05-12

**Authors:** Chloé Dimeglio, Marine Milhes, Jean-Michel Loubes, Noémie Ranger, Jean-Michel Mansuy, Pauline Trémeaux, Nicolas Jeanne, Justine Latour, Florence Nicot, Cécile Donnadieu, Jacques Izopet

**Affiliations:** 1 INSERM UMR1291—CNRS UMR5051, Toulouse Institute for Infectious and Inflammatory Diseases (INFINITy), 31300 Toulouse, France; izopet.j@chu-toulouse.fr; 2 Virology Laboratory, Centre Hospitalier Universitaire de Toulouse, Hôpital Purpan, 31300 Toulouse, France; ranger.no@chu-toulouse.fr (N.R.); mansuy.jm@chu-toulouse.fr (J.-M.M.); tremeaux.p@chu-toulouse.fr (P.T.); jeanne.n@chu-toulouse.fr (N.J.); latour.j@chu-toulouse.fr (J.L.); nicot.f@chu-toulouse.fr (F.N.); 3 Genotoul-Genome & Transcriptome—Plateforme Génomique (GeT-PlaGe), US INRAe 1426, 31326 Castanet-Tolosan, France; marine.milhes@inrae.fr (M.M.); cecile.donnadieu@inrae.fr (C.D.); 4 Institut de Mathématiques de Toulouse, Université de Toulouse, 31400 Toulouse, France; loubes@math.univ-toulouse.fr

**Keywords:** SARS-CoV-2, public health, statistical model, variant B.1.1.7

## Abstract

The spread of SARS-CoV-2 and the resulting disease COVID-19 has killed over 2.6 million people as of 18 March 2021. We have used a modified susceptible, infected, recovered (SIR) epidemiological model to predict how the spread of the virus in regions of France will vary depending on the proportions of variants and on the public health strategies adopted, including anti-COVID-19 vaccination. The proportion of SARS-CoV-2 variant B.1.1.7, which was not detected in early January, increased to become 60% of the forms of SARS-CoV-2 circulating in the Toulouse urban area at the beginning of February 2021, but there was no increase in positive nucleic acid tests. Our prediction model indicates that maintaining public health measures and accelerating vaccination are efficient strategies for the sustained control of SARS-CoV-2.

## 1. Introduction

The severe acute respiratory syndrome coronavirus 2 (SARS-CoV-2) that emerged in Wuhan, China, in December 2019 spreads mainly by sustained human-to-human transmission [1]. Its spread was so rapid that the WHO declared the resulting disease a pandemic [2]. Many countries opted for a strict lockdown in March 2020 to slow the epidemic and protect their health services. However, SARS-CoV-2 resumed its rampage in Europe, including France, at the end of summer 2020. The measures taken by several large cities to limit virus transmission have provided data that can be used to quantify the impact on virus proliferation of measures such as mask wearing, restricting access to public spaces, and curfew [3]. The French authorities established a new lockdown from 29 October to 28 November, which was followed by a gradual release under strict sanitary conditions, including a 6 p.m. curfew, together with the start of the COVID-19 vaccination campaign in early 2021 [4]. These vaccines can protect individuals from COVID-19 symptoms and induce population immunity, so reducing SARS-CoV-2 transmission [5,6,7,8]. However, a new SARS-CoV-2 variant, B.1.1.7 (GR/20I/501Y.V1), emerged in the southeast of England in October 2020 [9] and has spread to several countries including France since December 2020. Recent studies indicate that this new variant is up to 70% more readily transmissible than the original virus and is responsible for an increase in case numbers [10,11], although its real impact on virus dynamics in specific area remains elusive.

We have evaluated the impact of the increased proportion of SARS-CoV-2 variant B.1.1.7 in positive tests and ranked the impact of health measures such as physical distancing, mass testing, and vaccination on SARS-CoV-2 proliferation using data for the French city of Toulouse.

## 2. Materials and Methods

### 2.1. Statistical Model

Earlier models for SARS-CoV-2 were based on published positive cases and did not take into account the patients’ ages or an evolutive diffusion coefficient [12,13]. That is probably why the Johns Hopkins University predictive model underestimated the spread of the virus in Italy and overestimated its spread in France and the UK. Our model is a discretized version of a susceptible–infectious–recovered (SIR) model [14]. These compartmental models are well suited to studies of the spread of SARS-CoV-2 in different populations [15,16]. Our model includes a diffusion/transmission coefficient $R_{0}$ that varies with the likelihood of contagion, and two reduction coefficients $\hat{c}\;$ and $\hat{q}$ that describe the impact of public health measures on virus transmission. It also includes a parameter $p_{v}$ that reflects the prevalence of variant B.1.1.7 on the Toulouse urban area, plus a coefficient $\hat{i_{p}}$ that indicates how the proportion of variant influences the number of new infections. The model predicts how the SARS-CoV-2 virus would have evolved and projects the daily percentages of new positive cases. Then, the resulting data can be used to obtain a projection of SARS-CoV-2 seroprevalence in the Toulouse urban area.

We have used five variables $\left(S_{n},\;P_{n},\;Q_{n},\;I_{n},\;V_{n}\right)$, where $S_{n}$ is the number of healthy people who have not been vaccinated on day n, and $P_{n}^{i}$ is the number of undetected contagious carriers infected for i days $\left(1\leq i\leq N_{T}\right)$. Similarly, $Q_{n}^{i}$ is the number of detected contagious carriers infected for i days $\left(1\leq i\leq N_{T}\right)$ on day n. $I_{n}$ is the number of people who were immunized by an infection and $V_{n}$ is the number of people who were immunized by vaccination. We assume that the risk of reinfection by SARS-CoV-2 after a first infection or vaccination escape is negligible.

$N_{T}$ is the number of days a person is contagious and α is the percentage of the population tested each day. $R_{0}$ is the number of healthy people contacted and infected by a contagious person. We considered two $R_{0}$ figures: $R_{0}\left(V\right)$ for variant B.1.1.7 and $R_{0}\left(B\right)$ for the original SARS-CoV-2 virus. We assumed that they varied over time and peaked when the virus load was maximal: 7 days after the start of infection [17,18]. We also assumed that the number of days a person was contagious is equal to the time of infection i.e., 20 days [17,19]. For all $1\leq i\leq N_{T}$, $R_{0}^{i}=R_{0}.\;e^{-\frac{1}{2}{\left(\frac{i-7}{\sqrt{20}}\right)}^{2}}$.

N is the total population at the start of the epidemic phase,$\;\hat{i_{p}}$ is the multiplier for the spread of the UK variant $\left(0\leq \hat{i_{p}}\leq 1\right)$, $\hat{c}$ is the multiplier for the spread of the epidemic throughout public health restriction phases $\left(0\leq \hat{c}\leq 1\right)$, and $\hat{q}$ is the same multiplier during the quarantine period $\left(0\leq \hat{q}\leq 1\right)$. $\hat{c}$ and $\hat{q}$ are set at 1 when there is no restriction or quarantine. The lower the values of $\;\hat{c}$ or $\hat{q}$, the greater the constraint applied to halt the spread of the virus. Some values of $\hat{c}$ and the value of $\hat{q}$ have been estimated in previous work by correcting the values predicted by the model using real data collected by the Toulouse Virology Laboratory [3,20].

∀ n ∈ ⟦d+1,+∞⟧ $\hat{c}$ is defined as: $\hat{c}=\underset{c}{\mathrm{argmin}}{\left|{\hat{P}}_{n}-P_{n}\left(c\right)\right|}_{n\;\in \;⟦1,\;d⟧}$.

$\forall \;n\;\in \;⟦d_{1}+1,+\infty ⟧\;$ $\hat{i_{p}}$ is defined as $\;:\;\hat{i_{p}}=\underset{i_{p}}{\mathrm{argmin}}{\left|{\hat{P}}_{n}-P_{n}\left(i_{p}\right)\right|}_{n\;\in \;⟦1,\;d_{1}⟧}$.

We used data collected by the Toulouse Virology Laboratory from March 2020 to June 2020 to set $\hat{q}$ to 0.05 [20]. The values of $\hat{c}$ varied according to the public health restrictions implemented in the Toulouse area [3].

N  is given by Equation (1)

(1) $$N=S_{n}+P_{n}+Q_{n}+I_{n}+V_{n}$$

On transition from day n to day n+1, we have:

(2) $$\forall \;1\leq i\leq N_{T}-1,\;P_{n+1}^{i+1}=P_{n}^{i}\;\left(1-\alpha \right).$$

According to this equation, the number of undetected contagious carriers on day n+1 is the number of untested, undetected carriers who were infected but not detected on day n.

(3) $$P_{n+1}^{1}=\frac{S_{n}}{N}. [\underset{i}{\sum }P_{n}^{i}.(R_{0}^{i}\left(B\right).\left(1-p_{v}.\hat{i_{p}}\right)+R_{0}^{i}\left(V\right).p_{v}.\hat{i_{p}}).\hat{c}+\underset{i}{\sum }Q_{n}^{i}.(R_{0}^{i}\left(B\right).\left(1-p_{v}.\hat{i_{p}}\right)+R_{0}^{i}\left(V\right).p_{v}.\hat{i_{p}}).\hat{q}\;]$$

The above equation indicates that the number of new undetected contagious carriers on day n+1 is the number healthy people who were infected by undetected carriers at any stage of infection or by detected carriers at any stage of infection on day n.

(4) $$I_{n+1}=I_{n}+P_{n}^{N_{T}}+Q_{n}^{N_{T}}$$

Equation (4) indicates that the number of immunized people on day  n+1 corresponds to the number of people immunized on day n plus those people at the end of infection on day n, whether or not they were tested.

$Q_{n+1}^{1}=0\;$(no quarantine on day one, test results needed)

(5) $$\forall \;1\leq i\leq N_{T}-1,\;Q_{n+1}^{i+1}=Q_{n}^{i}+P_{n}^{i}\cdot \alpha$$

Equation (5) defines the number of detected contagious carriers on day n + 1 as the number of detected contagious carriers on day n plus the number of tested but undetected contagious carriers on day n.

Before 28 January 2021, we set: $V_{n}=0$

From 28 January 2021:

(6) $$V_{n+1}=V_{n}+794.$$

Equation (6) defines the number of vaccinated people on day n+1 as the number of people vaccinated and immunized on day n (two doses of vaccine) plus those given the second dose of vaccine 7 days before day n+1 [5]. This number was 794 in the Toulouse urban area, according to the Regional Health Agency [21].

We set $\;R_{0}\left(B\right)=2.2$ for the original SARS-CoV-2 virus, at its peak, based on a national and regional French study [22] and WHO international evaluations [23]. We added 0.4 to the basic reproduction number $R_{0}\left(B\right)$ to obtain the reproduction number $R_{0}\left(V\right)$ for the variant B.1.1.7 [24,25].

This section may be divided by subheadings. It should provide a concise and precise description of the experimental results, their interpretation, as well as the experimental conclusions that can be drawn.

### 2.2. Study Population

We estimated the initial model settings using data collected by the Toulouse Virology Laboratory (Table 1).

The total number of tests each day was 2500 at most. We assumed that 3.2% of the population of Toulouse urban area (about 1 million; source INSEE) had been infected with SARS-CoV-2 at the end of the first lockdown (11 May 2020) [26], which implies that there were close to 32,000 immune individuals, $I_{0},$ in mid-May. We also assumed that 20% of infections were asymptomatic [27,28]. The number of SARS-CoV-2 cases gradually increased from 21 July 2020 (time = 0 for the model). The date d corresponds to two periods (February 5 to February 12, 2021, and March 5 to 12 March 2021) for the evaluation of two health measures in addition to the 6 p.m. national curfew: the closure of non-food areas of over 20,000 m^2^ (from 31 January) and the closure of the banks in the Garonne river (from February 28). The date $d_{1}$ is the period 27 January to 1 February 2021.

The daily percentage of new SARS-CoV-2 cases was predicted using the initial parameters (Table 1). We used the number of cases on the previous day and two contagion parameters $(R_{0}^{i}\left(B\right))\;\mathrm{and}\;(R_{0}^{i}\left(V\right))\;$ that varied according to the strain of SARS-CoV-2 on the day of infection, and the constraints in force (quarantine, lockdown, or restrictions). We assumed that COVID-19 vaccination began on 1 January 2021. The theoretical efficacy of vaccination was set at 94%, as stated by the Pfizer trial [5], and we considered a subject to be immunized 7 days after the second dose (28 days after the first injection).

### 2.3. SARS-CoV-2 RNA Detection and Sequencing

Nasopharyngeal swab samples collected at two drive-through testing centers (Toulouse Purpan and Toulouse Rangueil sites) from 8 January 2021 to 7 February 2021 were tested using the ThermoFisher TaqPath RT-PCR assay (Scientific TaqPath COVID-19 Combo Kit, Thermo Fisher, Waltham, USA) after nucleic acid extraction using the MGI automated extraction system (MGI Easy Nucleic Acid Extraction Kit). All positive nasopharyngeal samples with cycle threshold (Ct) values below 28 (Ct ≤ 28 for N-gene) were tested using the Illumina CovidSeq test (COVIDSeq, Illumina, USA) on the Illumina Nova Seq 6000 Sequencing System (Genotoul platform, GeTPlaGe, Toulouse). SARS-CoV-2 sequences were analyzed using the Illumina DRAGEN COVIDSeq test pipeline.

## 3. Results

### 3.1. Evolution of the Proportion of the SARS-CoV-2 Variants

**Figure 1:**
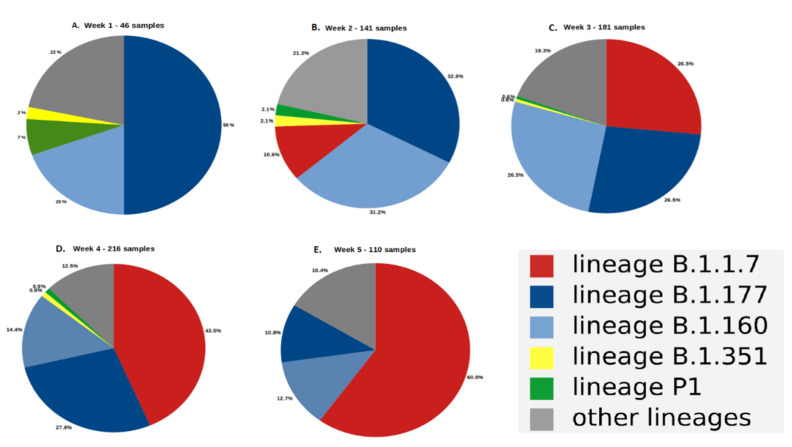
The circulating forms of SARS-CoV-2 on Toulouse urban area during January 2021 and early February. (**A**): week 1 (46 samples), (**B**): week 2 (141 samples), (**C**): week 3 (181 samples), (**D**): week 4 (216 samples), (**E**): week 5 (110 samples).

Sequencing of all the positive nasopharyngeal samples (Ct ≤ 28) showed that the main variants in the Toulouse urban area at the beginning of January were B.1.177 (GV/20E.EU1) with 51% of the total and B.1.160 (GH/20A.EU2) with 20% of the total (Figure 1A). The proportion of the B.1.177 variant decreased during January from 51% to 11% on 7 February (Figure 1E). The proportion of the B.1.160 variant varied between 31.2% and 12.7% during the same period. In contrast, the B.1.1.7 variant, which was not found during the first week in January (Figure 1A), appeared gradually (11% in the week from 11 January to 17 January 2021) (Figure 1B) and became the major form of the virus (60%) in the Toulouse urban area at the beginning of February (Figure 1E). Other notable variants, B.1.351 (GH/20H/501Y.V2, South Africa) and P1 (GR/20J/501Y.V3, Brazil), believed to be highly transmissible and less sensitive to neutralizing antibodies [29,30], appeared in early January 2021 but never accounted for more than 3% of the total in positive tests except for the first week of sequencing, which has few samples (Figure 1A).

### 3.2. Outlook for the Evolution of the SARS-CoV-2 Dynamics in the Urban Area of Toulouse

The match between the values predicted by the model and the values observed for 21 days over different split periods is given by a goodness-of-fit measure, R^2^ = 87%.

**Figure 2:**
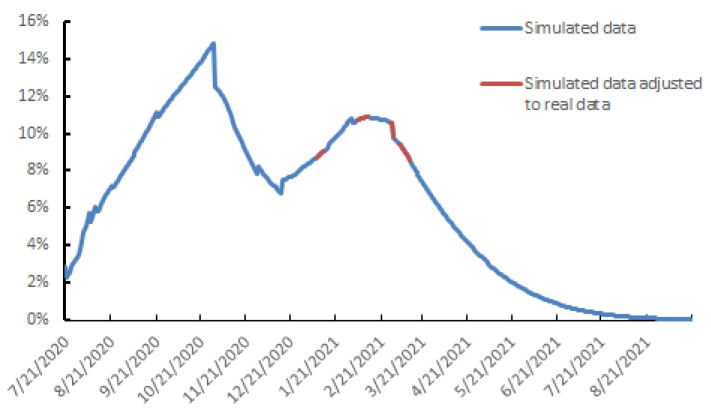
Daily spread of SARS-CoV-2 infection, 21 July 2020 to 5 September 2021.

A previous study showed that the 6 p.m. curfew imposed from 20 January 2021 reduced the circulation of SARS-CoV-2 among Toulouse inhabitants by 35% [31]. The average positive RT-PCR tests was 10.5% between 27 January and 1 February 2021 (Figure 2). We used the sequencing data to estimate how the proportion of SARS-CoV-2 variant B.1.1.7, $p_{v}$, changed during the month of January. The B.1.1.7 variant was the major variant (60%) circulating in the Toulouse urban area from 27 January to 1 February (Figure 1E). The hypotheses for its $R_{0}$ set out in Methods ($R_{0}=2.6)$ predicted that the percentage of new positive cases tested over this period would be 21.2% if the variant B.1.1.7 was responsible for 100% of SARS-CoV-2 infections. After correction based on the observed data for this interval, its estimated impact on virus dynamics $\hat{i_{p}}$ is about 2%.

Vaccination increased the proportion of the Toulouse urban area population immunized against SARS-CoV-2 after January 28. We also used the data for 5–12 February 2021 to assess adherence to the closure of large (>20,000 m^−2^) non-food areas. The 6 p.m. curfew should have increased the number of positive RT-PCR tests to 11–12% by mid-February. Instead, the positive tests plateaued at around 10.5%, when the constraint was greatest (37%). Using these data, and assuming that vaccination continued at the same pace, the percentage of daily new positive cases should slowly increase to 11% at the middle of March 2021 (Figure 2). The closure of banks in the Garonne on 28 February 2021, which made it possible to limit population groupings, provides us with the second set of data to fit our model over the period 5 March to 12 March 2021. The percentage of positive tests varied between 7.5% and 8% during this period, which corresponds to a 41% reduction in virus replication (Figure 2). These constraints plus continued vaccination should limit the percentage of positive tests to 10.9% on 13 February 2021. Then, the spread of SARS-CoV-2 should decrease to 7.8% positive tests on 15 March 2021 and 4.4% on 15 April (Figure 2). In parallel, the proportion of people immunized by natural infection or vaccination should increase from 10.1% on 13 February to 16.3% on 15 April.

### 3.3. The Parameters Influencing the Spread of SARS-CoV-2

Our model includes three parameters that influence the spread of the virus: public health measures (person–person barriers, closure of some public spaces, restrictions on store opening, curfews), mass testing, and vaccination. We have evaluated their impact in order to provide a rational basis for public health strategies. We focused on the period following the positive test peak, 14 February to 1 March 2021.

**Figure 3:**
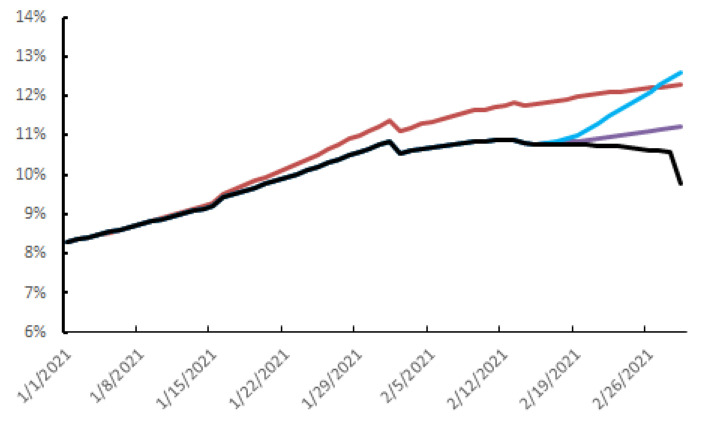
Daily spread of SARS-CoV-2 infection, 1 January 2021 to 1 March 2021 depending on the vaccine strategy and the release of public health measures or testing–tracing from 14 February 2021. Black curve: current situation, purple curve: reducing mass testing by half, blue curve: end of mass testing strategy, red curve: no vaccination strategy.

The barrier measures, testing–tracing–isolation and vaccination, decreased the positive tests from their peak on 13 February to 9.8% on 1 March 2021. Halving the testing capacity but continuing all the other measures would increase the spread of virus to give 11.2% positive tests on 1 March 2021 (purple curve, Figure 3). Similarly, completely stopping mass testing would increase the percentage of positive tests to 12.6% on the same date (blue curve, Figure 3). Without vaccination, but with all the other measures, would have increased the percentage of positive tests after 13 February to reach 12.3% on 1 March 2021 (red curve, Figure 3). Lifting all the barriers on 14 February 2021, even with the continued vaccination and mass testing, would result in contamination exploding to reach 59% on 1 March 2021.

## 4. Discussion

We have used a discretized SIR model to predict the spread of SARS-CoV-2 infections and evaluated the impact of the SARS-CoV-2 variant B.1.1.7, mass testing, vaccination, and other public health measures. By sequencing all the RT-PCR positive samples with Ct ≤28, we find that the distribution of SARS-CoV-2 strains changed in the Toulouse urban area from January 2021. The Brazilian (P1) and South African (B.1.1.351) variants did not spread, despite being highly transmissible; they accounted for a negligible fraction of the circulating virus. In contrast, the UK variant (B.1.1.7), which did not exist in early January, quickly became the major circulating strains of SARS-CoV-2 (60% at the beginning of February). This rapid growth is similar to that observed in United Kingdom and indicates its great capacity to adapt to its host [9]. However, there was not a comparable increase in the rate of positive tests, which should have occurred, given its assumed elevated $R_{0}$ [24]. We deduced that the impact of this variant on virus spreading was limited (2% at most), which would indicate that all the health measures and the testing–tracing–isolation policy are effective on this variant, in contrast to some reports [10,11]. These findings are consistent with those reported very recently [32]. An English study reported a theoretically higher rate of transmissibility for the B.1.1.7 variant than for the other variants but without changes in either severity or hospital dynamics. The study concluded that the health measures that are applied, including social distancing, vaccination, and lockdown, even regional, made it possible to keep the B.1.1.7 variant under control [32]. Our result also questions the hypothesis underlying the $R_{0}$ assigned to this variant; it accounts for increased transmissibility, which is not verified by the observed data. Of the three variants, B.1.1.7, B.1.351, and P1 harboring the N501Y mutation in the Spike protein, characterized by nasopharyngeal loads higher than that of the other strains (data not shown), only the B.1.1.7 variant became predominant. A recent study reported that B.1.1.7 dominates over another variant, B.1.351, in geographic areas where both variants co-circulate, and the B.1.1.7 was the first variant introduced in the population [33]. In the case of the Toulouse metropolitan area, this hypothesis seems difficult to sustain as the circulation of SARS-CoV-2 had already experienced several peaks before the first detection of the English variant and as there was a pre-existing dominant strain, the variant B.1.177. We could also consider that the B.1.1.7 variant could induce more asymptomatic infections than the other variants. This could explain its dominance without affecting the dynamics of positivity rates by RT-PCR tests. However, recent studies showed that there is no greater proportion of asymptomatic cases with the increase in the proportion of the B.1.1.7 variant among circulating strains of SARS-CoV-2 [32].

The second lockdown began on 29 October 2020 in France, and it has been gradually relaxed from 28 November with health measures to the epidemic restarting, including a nation-wide 6 p.m. curfew [31]. We find that the health measures have the greatest impact on the spread of SARS-CoV-2; the rate of positive tests would have increased 6-fold on 1 March if we had stopped all health measures on 14 February. Mass testing has a more moderate impact on virus replication dynamics; the positive test rate would have increased by 14% had we halved the number of tests and by 29% if we had stopped screening altogether. This more moderate impact could well be due to the maintenance of strong physical distancing measures. They contain the spread of the virus whatever the immune status. The vaccination policy pursued in the Toulouse urban area reduced the spread of SARS-CoV-2 by around 25%. As vaccination started at the end of 2020 in the Toulouse urban area, the first impact was not seen until 28 days later. There were signs that vaccination was having an effect on 14 February. Had vaccination begun earlier and more massively, these effects would probably have been more marked [34]. However, the positive test rate would have been rising after 13 February even without vaccination, and despite the testing and physical distancing. We have assumed that vaccinated people will not transmit SARS-CoV-2 to healthy unvaccinated people, but this is only an hypothesis as phase 3 clinical trials of COVID-19 vaccines were not designed to demonstrate the prevention of transmission [5,6,7]. Nevertheless, recent reports suggest that mRNA vaccines do block SARS-CoV-2 transmission. A study on nearly 1.2 million people, half of whom were vaccinated, confirmed that these vaccines protected 94% of patients against symptomatic forms of the disease [35]. It also indicates that the vaccine provides up to 90% protection against asymptomatic infections seven days after the second dose of vaccine [35]. Another study, conducted by the Sheba Medical Center in Israel on nearly 10,000 hospital staff frequently tested after vaccination, found that 85% of the 7214 members given their first dose of vaccine in January were protected 15 to 28 days later [36]. The overall reduction in asymptomatic infections was around 75%. Two other Israeli studies sought to assess the virus load in vaccinated people still carrying the virus [37,38]. They both concluded that vaccination significantly reduced the virus load in those who had become infected. This suggests that anti-SARS-CoV-2 vaccines greatly reduce virus excretion and, therefore, its transmission.

Our study has several limitations. The forecasts obtained with this SIR-type epidemiological model assume that its parameters remain stable over time. This is why we have chosen to work on local data, which we could configure precisely, and which have the quality of remaining rather stable over time. This is what allows us to obtain an 87% match between real data and simulated data. Parameter estimation in all mathematical models can lead to biased projections. We have attempted to overcome this problem for the parameters $\hat{c}\;$ and $\hat{q}$ that account for the impact of public health measures by correcting the predictions with observed data [3,20,31,34]. We also did this to estimate the impact of the variant B.1.1.7, $\hat{i_{p}}$, on virus spreading. The estimated virus proliferation rates resulting from the application of various public health measures also assume that the population continues to adhere to these measures over time. Obviously, estimating the impact of vaccination on virus spread assumes that the sociological constraints and the vaccination campaign are widely adhered to, especially among priority populations.

In conclusion, the emergence of the B.1.1.7 variant led to a rapid alteration in the distribution of circulating strains of SARS-CoV-2. The B.1.1.7 variant accounted for 60% of the circulating SARS-CoV-2 viruses in the Toulouse urban area at the beginning of February, without any increase in the positive test rate. Currently, the B.1.1.7 variant accounts for over 90% of positive tests while the positive test rate is still decreasing. Public health measures and testing–tracing–isolation policies appear to be effective against this variant, as is vaccination. Maintaining public health measures and accelerating vaccination must both be employed to keep SARS-CoV-2 under control.

## Figures and Tables

**Table 1:** Model initial parameters.

Age Group	% In Toulouse Population. (Source INSEE)	A (Based on Toulouse Data)	$N_{T}$
<15 years old	14.8%	10.2%	20
15–60 years old	68.2%	61.58%	20
60–74 years old	10.4%	10.68%	20
>74 years old	5.5%	17.54%	20

## Data Availability

The datasets used and/or analysed during the current study are available from the corresponding author on request.
